# Association of DHA Concentration in Human Breast Milk with Maternal Diet and Use of Supplements: A Cross-Sectional Analysis of Data from the Japanese Human Milk Study Cohort

**DOI:** 10.1093/cdn/nzaa105

**Published:** 2020-06-15

**Authors:** Hiroshi M Ueno, Satoshi Higurashi, Yuzuka Shimomura, Ryota Wakui, Hiroaki Matsuura, Makoto Shiota, Hiroaki Kubouchi, Jun-ichi Yamamura, Yasuhiro Toba, Toshiya Kobayashi

**Affiliations:** Research and Development Department, Bean Stalk Snow Co., Ltd., Kawagoe, Japan; Research and Development Department, Bean Stalk Snow Co., Ltd., Kawagoe, Japan; Milk Science Research Institute, Megmilk Snow Brand Co., Ltd., Kawagoe, Japan; Milk Science Research Institute, Megmilk Snow Brand Co., Ltd., Kawagoe, Japan; Milk Science Research Institute, Megmilk Snow Brand Co., Ltd., Kawagoe, Japan; Milk Science Research Institute, Megmilk Snow Brand Co., Ltd., Kawagoe, Japan; Milk Science Research Institute, Megmilk Snow Brand Co., Ltd., Kawagoe, Japan; Research and Development Department, Bean Stalk Snow Co., Ltd., Kawagoe, Japan; Research and Development Department, Bean Stalk Snow Co., Ltd., Kawagoe, Japan; Research and Development Department, Bean Stalk Snow Co., Ltd., Kawagoe, Japan

**Keywords:** docosahexaenoic acid, human breast milk, Japan, maternal diet, supplementation

## Abstract

**Background:**

DHA (22:6n–3) is essential for neurodevelopment in children, and its concentration in human breast milk is historically high in Japan. Dietary patterns in Japan might affect the fatty acid (FA) composition among lactating mothers.

**Objectives:**

This study aimed to characterize the composition of milk FAs and to identify any dietary and sociodemographic factors associated with the variability of DHA concentration in breast milk in the Japanese population.

**Methods:**

This cross-sectional study was performed as part of the Japanese Human Milk Study. Milk FAs were analyzed by GC at 1–6 mo postpartum, and maternal diet was estimated using an FFQ, including 11 types and cooking methods of seafoods, and the use of DHA supplements. The association of milk DHA with maternal diet and sociodemographic factors was investigated.

**Results:**

Milk FA concentrations were measured in 78 mothers, including 24 who routinely used DHA supplements. The DHA concentration in milk (overall median: 0.62%; IQR: 0.47%–0.78%) was higher in women who took DHA supplements than in women who had never used DHA supplements (0.74%compared with 0.55%; *P *= 0.011). A linear regression model showed the association of milk DHA concentration with maternal dietary intake of grilled fish (β ± SE: 0.006 ± 0.003; standardized β: 0.234; *r*^2^ = 0.232, *P *= 0.036) after adjustment for DHA supplementation status, maternal and infant age, maternal BMI, and infant birth weight. Other FA concentrations were consistent, whereas caproic acid (6:0), undecylic acid (11:0), pentadecylic acid (15:0), palmitoleic acid (16:1n–7), and vaccenic acid (18:1n–7) varied by DHA supplementation status.

**Conclusions:**

The DHA concentration in human milk may be influenced by maternal grilled fish consumption and frequent DHA supplementation in lactating Japanese women. Milk DHA concentrations may reflect a dietary habit in Japanese mothers.

This trial was registered at www.umin.ac.jp/ctr as UMIN000015494.

## Teaser

Milk docosahexaenoic acid and maternal diet in Japan: grilled fish intake and frequent use of supplements may influence milk fatty acid composition.

## Introduction

Breastfeeding benefits both children and their mothers. For mothers, breastfeeding can prevent breast cancer, improve birth spacing, and reduce a woman's risk of diabetes and ovarian cancer (1).

Although the composition of human milk varies from woman to woman and over time, human milk fat provides almost 50% of the energy intake in young infants. Human milk fat is also important for the provision of essential fatty acids (FAs), lipid-soluble vitamins, and the bioactivity of specific components (2). Concentrations of FAs in breast milk vary widely according to maternal lipid intake, and there is a possibility that poor FA intake can alter neurological development in breastfed infants (3). PUFAs are of increasing interest because of the dependence of humans on dietary sources of n–3 PUFAs and the high amounts of DHA (22:6n–3) in the brain and retina. Worldwide, the mean concentration of DHA in human breast milk has been calculated to be 0.3%, although this figure varies widely according to geographic location and local environmental factors (4). Moreover, a study in a large Canadian birth cohort indicated natural variation in the FA composition of human milk in relation to dietary, genetic, sociodemographic, and health factors (5). DHA concentrations in human milk are high in Japan, the Philippines, and South Korea, but low in the United States and Canada (6, 7). Furthermore, the composition of FAs, including DHA, in human milk has been found to vary between river/lake, coastal, and inland regions of China, where the types of aquatic food consumed show regional variation (8).

In Japan, there has been a change in dietary patterns of fish and meat intake, resulting in some effects on the intake of DHA and EPA (20:5n–3) as well as arachidonic acid (20:4n–6), particularly in the young population (9). In addition, DHA supplements are becoming increasingly popular in pregnant and lactating women in East Asian countries, including China (10) and South Korea (7), in the belief that they will increase the DHA concentration in breast milk. However, the significance of dietary patterns including the manner of seafood consumption and use of supplements and their relevance in terms of DHA in human milk in populations with historically high DHA consumption are unclear.

The present study aimed to describe the composition of FAs in human milk and to investigate the relation of these FAs (in particular DHA) with dietary patterns (particularly the use of DHA supplements and seafood intake including cooking methods and type of seafood) in lactating women in Japan. We also sought possible associations between milk DHA and sociodemographic, birth-related, and environmental factors.

## Methods

### Participants and milk samples

This study was performed as a cross-sectional study in the early phase of the Japanese Human Milk Study cohort (11, 12). Healthy mothers and their infants were recruited from medical institutions throughout Japan from February 2015 to June 2017. Women were included in the study if they were healthy and lactating, of Japanese ethnicity and living in Japan, had singleton infants who were healthy at 0–6 mo postpartum, and were willing to collect milk samples and complete a dietary survey according to the instructions of the study protocol via telephone and documented instructions. The study participants were asked to collect milk samples into breast milk storage bags after breastfeeding (using a breast pump), once a day, for 7 d. The milk storage bags and manual breast pump (Yanase Waichi) were supplied by the study group at the registration of the study. Upon their arrival, we first pooled the 7 samples together, then conducted biochemical analyses including FAs, macronutrients, and energy composition. The milk samples were stored at −80°C until analysis. Milk samples were assessed in this study if ≥50 mL was available for analysis without affecting the amount of milk required for the analyses performed in the Japanese Human Milk Study. If a mother had >1 milk sample available at any of the assessments performed at 2-monthly intervals over 6 mo, the most recent eligible sample was included in the study. To mitigate unexpected influence of pharmaceuticals and medical conditions on the composition of human milk, women were excluded if they were taking medication or deemed unsuitable for participation in the study in the opinion of the investigators (e.g., if there was a recent history of microbial infection that could be transmitted via a milk sample).

### Analyses of milk macronutrient, energy, and FA composition

Before analysis, the milk samples were thawed in a water bath maintained at a temperature of 37–40°C and homogenized. Macronutrient and energy analyses were performed using a mid-infrared transmission spectroscopy device (Miris Human Milk Analyzer, Miris) developed for in-hospital analysis of human milk.

The lipid content was extracted from the milk using a modification of the method described by Folch et al. (13) with ∼15 mL milk-chloroform/methanol (2:1, vol:vol) solvent mixture (1:2, vol:vol) containing 1 g NaCl. Aliquots of the extract were determined gravimetrically after evaporation of the solvent. The FAs were analyzed according to the literature with minor modifications (14). Briefly, 25 mg lipid extract was subjected to derivatization of FAME, which was prepared with BF_3_:methanol (14%, wt:vol) and extracted into hexane. The measurements were performed using an Agilent 7890B Series Gas Chromatograph system interfaced with a flame ionization detector (Agilent Technologies). An SP-2560 column (100 m × 0.25 m × 0.20 μm; Sigma-Aldrich) was used and calibrated against a standard cocktail (Supelco 37 component FAME mix, Sigma-Aldrich). The profile of the FAs in breast milk was detected by GC and calculated with FAMEs using the internal standard method (13:0 and 17:0 FAMEs). One microliter of the sample was injected with the He carrier gas set at a flow rate of 1.0 mL/min with a split ratio of 100:1 and constant control of flow. The temperatures of the injector and detector were set at 250°C. The column oven was programmed at 180°C initially, maintained at this temperature for 45 min, increased by 2°C/min for 5 min, increased by 3°C/min to 220°C, and maintained at this temperature for a total of 33 min. The FAMEs were identified according to their retention times using qualified standards, and the proportions of FAs were calculated by quantification of the peak areas. All experimental procedures were performed in duplicate.

### FFQ

Maternal diet during lactation was estimated using an FFQ. All study participants completed the Brief-type self-administered Diet History Questionnaire (BDHQ) at the time of the milk collection for this study. The BDHQ is a short version of the Diet History Questionnaire (DHQ) and can be used easily in the clinical setting, including in pregnant women (15, 16). The BDHQ is a fixed-portion questionnaire that assesses dietary intake during the previous month. The unadjusted and energy-adjusted intakes for energy, nutrients, and foods (per day and 1000 kcal) measured by the DHQ and the BDHQ were calculated using an ad hoc computer algorithm based on the standard tables for food composition in Japan. For this study, we used the energy-adjusted intakes for further analyses based on the BDHQ validation study (15, 17). Types of fish included shellfish, fish with edible bones, canned tuna and bonito, dried fish, fatty fish, and fat-free fish. Preparation methods included eating raw, grilling, boiling, and deep-frying. Maternal PUFA intake was evaluated according to the Dietary Reference Index in Japan (18).

Another dietary questionnaire was administered to obtain information about daily DHA supplement intakes and frequency of supplementation. The frequency of supplement consumption was categorized as never, infrequent (1–4 d/wk), frequent (5–6 d/wk), daily, or supplemented but not recorded.

### Sociodemographic, anthropometric, and birth-related questionnaires

Further, a questionnaire that included queries on both mother and infant was self-administered by participants to obtain information on sociodemographic factors (age, maternal education, and household income), anthropometrics (maternal current and prepregnancy BMI, infant birth weight and length), feeding methods, and birth-related environmental factors (delivery, gestation, parity, sex) for analysis.

### Statistical analysis

The scarcity of quantitative data on FA content in lactating women who used DHA supplements precluded a power calculation in this exploratory study. The sample size was initially set at 20 subjects/group (never-supplemented and supplemented populations for DHA intake), according to the estimated feasibility of recruitment at each participating study center over a 2-y period.

Descriptive statistics were used to describe the study participants’ characteristics. In view of the nonnormal distribution (tested graphically and by the Shapiro–Wilk test), continuous variables are presented as medians and IQRs. Differences in characteristics between supplement users and never-users were investigated using the Mann–Whitney *U* test owing to unequal variance. Linear regression analyses were performed to identify significant predictors of the DHA content in milk. We used crude univariate linear regression analyses to explore the associations of milk DHA concentration with dietary, sociodemographic, anthropometric, and birth-related factors. Dietary factors that were significantly associated with milk DHA concentration in the crude analyses were further investigated in multivariate linear regression models, which were adjusted for maternal and infant age, maternal current BMI, and infant birth weight, to identify independent determinants of milk DHA concentration. Statistical analyses were performed using SPSS Statistics version 26.0 (IBM Corp.) and R version 3.2.1 (R Foundation for Statistical Computing). A *P* value < 0.05 was considered statistically significant.

### Ethics

The study protocol was approved by the internal review board of Fukuda Clinic (approval number IRB20140621-03) and registered in the Japanese Clinical Trials Registry (UMIN000015494). All study procedures were performed in accordance with the principles of the 1975 Declaration of Helsinki, as revised in 1983. All study participants provided written informed consent at the time of their enrollment in the Japanese Human Milk Study.

## Results

### Subject characteristics and maternal diet

A total of 78 mother–infant dyads participated in the study. Twenty-four mothers reported the use of DHA supplements; 6 mothers reported a low frequency of use (1–4 d/wk), 4 reported frequent use (5–6 d/wk), 9 reported daily use, and 5 did not report their frequency of use. **Supplemental Figure 1** shows the disposition of the study participants and their flow through the study. The median maternal age was 31 y, the median gestational period was 39 wk, and the median infant birth weight was 3100 g (**Table 1**). The median infant age was 92 d for all study participants, 98 d for infants born to never-users, and 72 d for infants born to supplement users. No significant difference was observed in the use of DHA supplements. The median energy-adjusted dietary n–3 PUFA and DHA intakes were not significantly different between never-users and supplement users (*P *= 0.363 for n–3 PUFAs; *P *= 0.335 for DHA).

**FIGURE 1:**
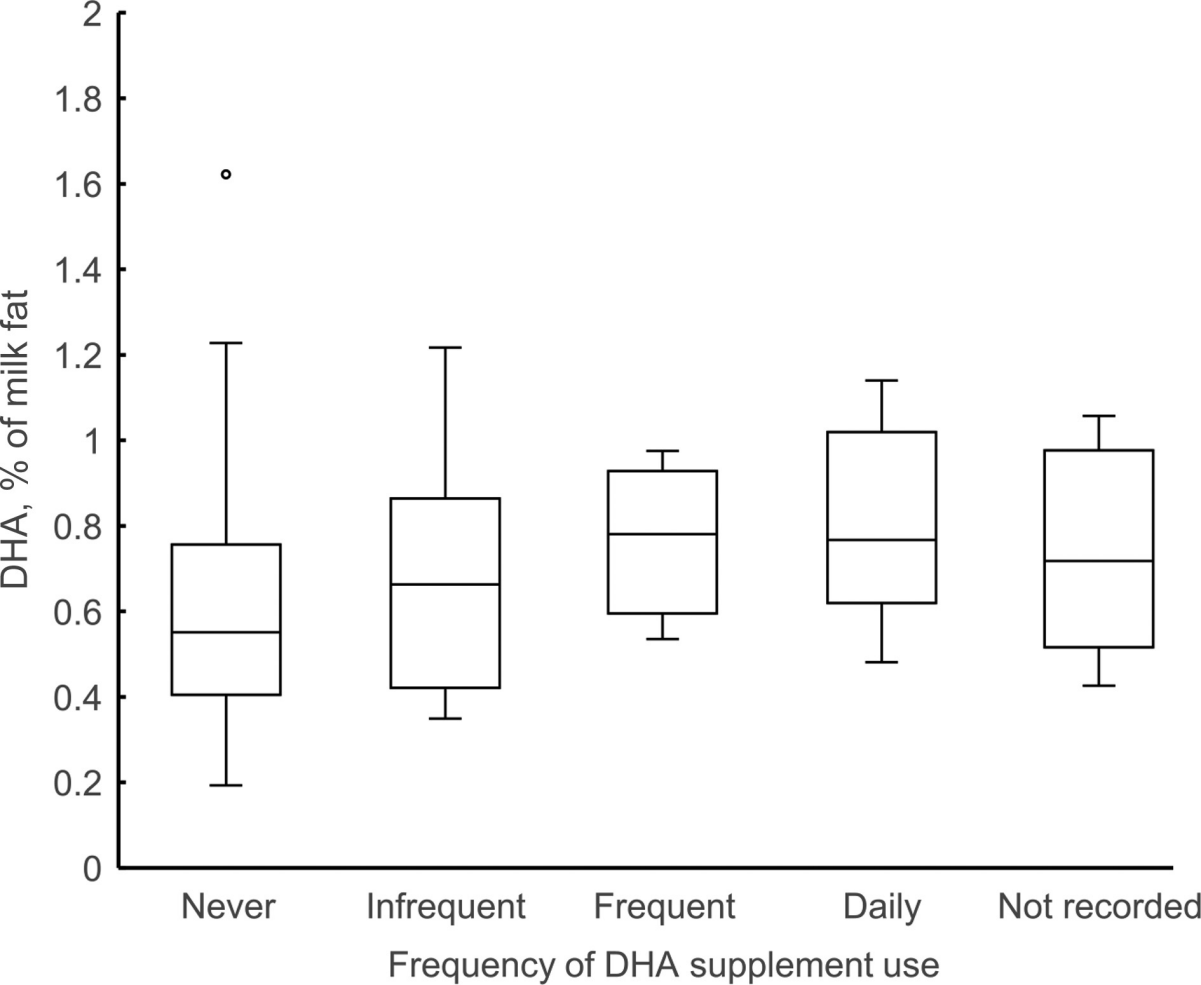
Milk DHA concentration in relation to the frequency of DHA supplementation. The frequency of supplement consumption was categorized as never, infrequent (1–4 d/wk), frequent (5–6 d/wk), daily, or supplemented but not recorded. The box plot shows the lower (quartile 1) and upper (quartile 3) quartiles and the median, bars represents upper and lower values within 1.5 × interquartile ranges, and small circle shows an outlier.

**TABLE 1:** Maternal and infant characteristics^1^

		DHA supplement use		
	All participants (*n* = 78)	No (*n* = 54)	Yes (*n* = 24)	*P* value
Maternal characteristics				
Age, y	31 [28–35]	30 [28–34]	31 [28–35]	0.522
BMI, kg/m^2^				
Current	20.9 [19.7–21.9]	21.2 [19.8–22.2]	20.9 [19.2–21.8]	0.506
Prepregnancy	20.1 [18.9–21.6]	20.2 [19.1–21.8]	19.9 [18.7–21.3]	0.488
Education				0.977
JHS/HS/others	17 (21.8)	12 (22.2)	5 (20.8)	
Some college/technical	25 (32.1)	17 (31.5)	8 (33.3)	
4-y college/graduate degree	36 (46.2)	25 (46.3)	11 (45.8)	
Household income, JPY/y				0.871
<1,999,999	0 (0.0)	0 (0.0)	0 (0.0)	
2,000,000–3,999,999	20 (25.6)	14 (25.9)	6 (25.0)	
4,000,000–5,999,999	23 (29.5)	15 (27.8)	8 (33.3)	
6,000,000–7,999,999	22 (28.2)	17 (31.5)	5 (20.8)	
8,000,000–9,999,999	6 (7.7)	4 (7.4)	2 (8.3)	
<10,000,000	2 (2.6)	1 (1.9)	1 (4.2)	
Maternal nutrient intake from diet				
Energy, kcal/d	1729.2 [1471.1–1961.8]	1677.6 [1488.4–1961.8]	1734.2 [1429.5–1931.1]	0.762
Fat, g/1000 kcal	33.1 [30.1–36.8]	33.1 [29.3–36.5]	33.1 [31.5–37.0]	0.475
SFA, g/1000 kcal	9.36 [8.17–10.93]	9.20 [8.17–11.15]	9.56 [8.24–10. 42]	0.991
MUFA, g/1000 kcal	11.83 [10.73–12.74]	11.77 [10.39–12.87]	12.18 [11.14–12.65]	0.386
PUFA, g/1000 kcal	7.47 [6.44–8.32]	7.33 [6.33–8.27]	7.80 [7.20–8.74]	0.150
n–3 PUFA, g/1000 kcal	1.41 [1.21–1.67]	1.38 [1.14–1.65]	1.48 [1.28–1.77]	0.363
22:6n–3 (DHA), mg/1000 kcal	235.9 [183.6–352.0]	225.1 [181.9–351.5]	265.4 [194.6–367.0]	0.335
n–6 PUFA, g/1000 kcal	6.08 [5.16–6.73]	5.93 [5.07–6.54]	6.36 [5.74–6.88]	0.144
Maternal fish intake, cooking method				
Grilled, g/1000 kcal	16.76 [9.53–23.95]	16.88 [9.73–23.95]	15.89 [5.90–23.99]	0.577
Raw, g/1000 kcal	8.31 [4.98–15.93]	8.90 [5.23–15.93]	7.02 [4.80–15.53]	0.713
Boiled, g/1000 kcal	17.48 [8.42–44. 82]	16.28 [8.42–42.51]	19.39 [7.53–45.28]	0.854
Deep-fried, g/1000 kcal	5.74 [0.00–9.96]	5.77 [0.00–9.27]	5.14 [0.00–11.11]	0.904
Maternal seafood intake				
Shellfish, g/1000 kcal	4.12 [3.11–7.13]	4.00 [3.11–7.01]	4.44 [3.09–7.40]	0.858
Fish with edible bones, g/1000 kcal	2.69 [0.00–5.11]	2.08 [0.00–4.61]	3.33 [2.22–5.93]	0.053
Canned tuna and bonito, g/1000 kcal	1.76 [0.00–2.76]	1.72 [0.00–2.47]	1.82 [0.68–3.35]	0.324
Dried fish, g/1000 kcal	6.66 [3.66–11.31]	6.50 [3.66–9.26]	6.97 [3.55–13.91]	0.820
Oily fish, g/1000 kcal	7.23 [4.02–10.35]	7.02 [4.02–10.70]	7.54 [4.27–9.35]	0.983
Oil-free fish, g/1000 kcal	6.48 [3.93–9.02]	6.64 [3.98–9.46]	5.32 [3.68–8.33]	0.526
Seaweed, g/1000 kcal	3.09 [1.86–7.13]	3.04 [1.87–6.17]	4.54 [1.76–7.69]	0.363
DHA supplement use	24 (30.8)			
Never		54 (100.0)		
Infrequent (1–4 d/wk)			6 (25.0)	
Frequent (5–6 d/wk)			4 (16.7)	
Daily			9 (37.5)	
Frequency not recorded			5 (20.8)	
Birth and infant characteristics				
Cesarean delivery	11 (14.1)	9 (16.7)	2 (8.3)	0.486
Gestational period, wk	39 [38–40]	39 [38–40]	39 [39–40]	0.560
Age, d	92 [55–123]	98 [60–135]	72 [48–114]	0.122
Birth length, cm	50 [48–51]	49 [48–51]	50 [48–51]	0.764
Birth weight, g	3100 [2871–3366]	3154 [2930–3390]	3046 [2793–3355]	0.258
Sex, male	43 (55.1)	29 (53.7)	14 (58.3)	0.807
Parity, *n*	2 [1–2]	2 [1–2]	2 [1–2]	0.847
Breastfeeding characteristics at sample collection				
Exclusive breastfeeding	71 (91.0)	48 (88.9)	23 (95.8)	0.427

^1^Values are median [IQR] for continuous variables with skewed distribution and *n* (%) for categorical variables, unless otherwise indicated. Differences in variables according to frequency of use of DHA supplementation were examined using the Mann–Whitney *U* test. Dichotomous variables were compared using Fisher's exact test. HS, high school; JHS, junior high school; JPY, Japanese Yen.

### Milk macronutrients and FA profiles

The median total lipid content was 3.2 g/100 mL, and was consistent across the entire study population, never-users, and supplement users. **Table 2** presents data on other macronutrients, energy, and the composition of 31 identified and other FAs. The median DHA content in the total study population was 0.62% (IQR: 0.47%–0.78%) and was higher in supplement users (0.74%; IQR: 0.60%–0.90%) than in never-users (0.55%; IQR: 0.41%–0.76%) (*P *= 0.011). In contrast, the median dihomo-γ-linolenic acid (20:3n–6) content was higher in never-users than in supplement users (0.25% compared with 0.21%; *P *= 0.051). Total SFA concentrations were consistent, and total MUFA concentrations slightly varied across the populations (never-users compared with supplement users, 38.30% compared with 39.49%; *P *= 0.060). Significant differences in caproic acid (6:0), undecylic acid (11:0), and pentadecylic acid (15:0) contents were found between never-users and supplement users (*P *< 0.05; 0–0.05% high in never-users). The median n–7 MUFA content was higher in supplement users than in never-users, as were the palmitoleic acid (16:1n–7) content (2.39% compared with 2.09%; *P *= 0.004) and vaccenic acid (18:1n–7) content (1.83% compared with 1.74%; *P *= 0.010).

**TABLE 2:** Macronutrients, energy, and fatty acid composition in breast milk^1^

		DHA supplement use		
	All participants (*n* = 78)	No (*n* = 54)	Yes (*n* = 24)	*P* value
Macronutrients and energy				
Fat, g/100 mL	3.2 [2.5–4.0]	3.2 [2.5–4.0]	3.1 [2.3–3.9]	0.332
Crude protein, g/100 mL	1.0 [0.9–1.1]	1.0 [0.9–1.1]	1.0 [1.0–1.1]	0.948
True protein, g/100 mL	0.8 [0.7–0.9]	0.8 [0.7–0.9]	0.8 [0.8–0.9]	0.996
Carbohydrate, g/100 mL	7.3 [7.2–7.5]	7.3 [7.2–7.5]	7.4 [7.3–7.5]	0.270
Total solid, g/100 mL	11.8 [11.0–12.4]	11.9 [11.0–12.4]	11.5 [10.9–12.4]	0.488
Energy, kcal/100 mL	63 [56–71]	64 [57–71]	61 [56–69]	0.426
Fatty acid composition				
Total SFAs, %	40.76 [39.18–42.67]	41.17 [39.38–42.87]	39.99 [38.92–42.54]	0.183
6:0	0.06 [0.00–0.50]	0.07 [0.00–0.52]	0.02 [0.00–0.06]	0.028
8:0	0.25 [0.21–0.35]	0.24 [0.21–0.34]	0.27 [0.21–0.37]	0.705
10:0	1.39 [1.24–1.57]	1.37 [1.24–1.57]	1.42 [1.23–1.60]	0.888
11:0	0.01 [0.01–0.01]	0.01 [0.01–0.01]	0.01 [0.00–0.01]	0.013
12:0	5.27 [4.36–6.18]	5.26 [4.36–6.08]	5.39 [4.33–6.48]	0.948
13:0	0.02 [0.02–0.03]	0.02 [0.02–0.03]	0.02 [0.01–0.03]	0.232
14:0	5.00 [4.24–5.89]	5.07 [4.26–5.93]	4.85 [4.01–5.79]	0.417
15:0	0.22 [0.19–0.27]	0.23 [0.21–0.28]	0.21 [0.19–0.22]	0.031
16:0	21.33 [20.14–22.06]	21.29 [20.30–22.01]	21.40 [19.11–22.12]	0.649
17:0	0.26 [0.24–0.30]	0.27 [0.25–0.30]	0.25 [0.23–0.29]	0.147
18:0	6.26 [5.82–6.68]	6.28 [5.89–6.68]	6.20 [5.61–6.68]	0.649
20:0	0.19 [0.18–0.20]	0.19 [0.17–0.20]	0.19 [0.18–0.21]	0.803
22:0	0.07 [0.06–0.08]	0.07 [0.06–0.09]	0.07 [0.06–0.08]	0.247
23:0	0.07 [0.06–0.09]	0.07 [0.06–0.09]	0.08 [0.05–0.09]	0.812
Total MUFAs, %	38.93 [37.25–40.80]	38.30 [36.70–39.91]	39.49 [38.10–41.36]	0.060
14:1	0.14 [0.11–0.16]	0.14 [0.11–0.17]	0.13 [0.11–0.15]	0.238
16:1n–7 (palmitoleic acid)	2.17 [1.96–2.47]	2.09 [1.84–2.36]	2.39 [2.13–2.52]	0.004
17:1	0.17 [0.15–0.19]	0.17 [0.15–0.19]	0.17 [0.16–0.20]	0.778
18:1n–9 (oleic acid)	34.02 [32.41–35.95]	33.59 [32.38–35.26]	34.55 [32.96–36.27]	0.190
18:1n–7	1.76 [1.65–1.89]	1.74 [1.63–1.84]	1.83 [1.75–1.92]	0.010
20:1	0.42 [0.37–0.47]	0.41 [0.37–0.47]	0.44 [0.39–0.50]	0.127
22:1n–9	0.07 [0.06–0.08]	0.07 [0.06–0.08]	0.07 [0.06–0.09]	0.404
Total PUFAs, %	16.49 [15.75–17.97]	16.68 [15.82–17.64]	16.38 [15.39–18.69]	0.737
Total n–6 PUFAs, %	14.14 [13.21–15.22]	14.22 [13.50–15.12]	13.96 [12.94–15.76]	0.634
18:2 (linoleic acid)	13.22 [12.19–14.32]	13.24 [12.57–14.25]	13.01 [12.15–14.64]	0.681
18:3 (γ-linolenic acid)	0.09 [0.07–0.12]	0.10 [0.07–0.12]	0.08 [0.06–0.11]	0.163
20:2	0.23 [0.21–0.25]	0.24 [0.21–0.25]	0.23 [0.21–0.26]	0.468
20:3 (dihomo-γ-linolenic acid)	0.24 [0.21–0.28]	0.25 [0.22–0.28]	0.21 [0.19–0.27]	0.051
20:4 (arachidonic acid)	0.38 [0.34–0.41]	0.37 [0.34–0.41]	0.40 [0.33–0.41]	0.618
Total n–3 PUFAs, %	2.48 [2.16–2.92]	2.44 [2.15–2.84]	2.57 [2.32–2.95]	0.242
18:3 (α-linolenic acid)	1.45 [1.30–1.70]	1.44 [1.31–1.70]	1.47 [1.19–1.69]	0.991
20:3	0.05 [0.03–0.05]	0.05 [0.04–0.05]	0.04 [0.00–0.05]	0.212
20:5 (EPA)	0.15 [0.11–0.23]	0.16 [0.10–0.24]	0.15 [0.11–0.19]	0.837
22:5 (docosapentaenoic acid)	0.19 [0.16–0.24]	0.19 [0.16–0.25]	0.20 [0.17–0.23]	0.965
22:6 (DHA)	0.62 [0.47–0.78]	0.55 [0.41–0.76]	0.74 [0.60–0.90]	0.011
Others, %	3.10 [2.68–3.51]	3.18 [2.68–3.63]	2.98 [2.65–3.32]	0.213
n–6/n–3	5.74 [4.94–6.55]	5.81 [4.94–6.82]	5.48 [4.98–6.06]	0.194

^1^Values are medians [IQRs]. The effects of DHA supplementation were examined using the Mann–Whitney *U* test.

### Regression analyses

**Table 3** presents the influence of individual variables on univariate and multivariate linear regression analyses of DHA in breast milk. In the crude univariate models, maternal dietary intake of grilled fish (β ± SE: 0.006 ± 0.003; standardized β: 0.267; *r*^2^ = 0.071, *P *= 0.018) and infant age (β ± SE: −0.001 ± 0.001; standardized β: −0.260; *r*^2^ = 0.068, *P *= 0.022) predicted milk DHA concentration. The frequency of supplement use [never, infrequent (1–4 d/wk), frequent (5–6 d/wk), daily] was associated with milk DHA concentration (β ± SE: 0.067 ± 0.030; standardized β: 0.259; *r*^2^ = 0.067, *P *= 0.027), and the fixed effect of DHA supplementation was a significant factor (β ± SE: 0.144 ± 0.064; standardized β: 0.248; *r*^2^ = 0.062, *P *= 0.028). Other variables related to DHA intake, including the type of fish, method used to cook fish, and estimated DHA consumption recorded by the BDHQ, were not significantly associated with milk DHA concentrations. Based on the results of the univariate models, we chose maternal dietary intake of grilled fish and the fixed effect of DHA supplementation as the potential confounders for milk DHA concentrations. A multivariate model predicted that milk DHA concentration was associated with maternal dietary intake of grilled fish (β ± SE: 0.006 ± 0.003; standardized β: 0.234; *r*^2^ = 0.232, *P *= 0.036) after adjustment for the use of DHA supplements, maternal and infant age, maternal current BMI, and infant birth weight.

**TABLE 3:** Associations between milk DHA concentration, dietary fatty acids, and routine DHA supplementation^1^

	β	(95% CI); SE	Standardized β	*r* ^2^	*P* value
Crude univariate models					
Maternal use of DHA supplements					
Use of DHA supplements	0.144	(0.016, 0.273); 0.064	0.248	0.062	0.028
Frequency of DHA supplementation	0.067	(0.008, 0.126); 0.030	0.259	0.067	0.027
Maternal dietary intake, nutrients					
Fat	0.011	(−0.002, 0.024); 0.007	0.187	0.035	0.101
n–3 PUFAs	0.109	(−0.050, 0.269); 0.080	0.155	0.024	0.175
DHA	0.000	(−0.000, 0.001); 0.000	0.079	0.006	0.494
Maternal seafood intake, cooking method					
Grilled fish	0.006	(0.001, 0.012); 0.003	0.267	0.071	0.018
Raw fish	0.005	(−0.002, 0.012); 0.003	0.172	0.030	0.132
Boiled fish	−0.001	(−0.003, 0.002); 0.001	−0.057	0.003	0.623
Deep-fried fish	−0.004	(−0.012, 0.004); 0.004	−0.110	0.012	0.339
Maternal seafood intake, fish type					
Shellfish	0.006	(−0.006, 0.018); 0.006	0.118	0.014	0.302
Fish with edible bones	0.002	(−0.006, 0.010); 0.004	0.052	0.003	0.650
Canned tuna and bonito	0.010	(−0.019, 0.040); 0.015	0.080	0.006	0.486
Dried fish	0.003	(−0.004, 0.011); 0.004	0.095	0.009	0.410
Oily fish	0.000	(−0.009, 0.009); 0.005	−0.001	0.000	0.995
Oil-free fish	−0.002	(−0.012, 0.009); 0.005	−0.034	0.001	0.767
Seaweed	0.005	(−0.007, 0.017); 0.006	0.100	0.010	0.385
Maternal characteristics					
Age	0.000	(−0.014, 0.014); 0.007	−0.006	0.000	0.962
Current BMI	−0.011	(−0.039, 0.017); 0.014	−0.092	0.008	0.429
Prepregnancy BMI	−0.006	(−0.027, 0.016); 0.011	−0.062	0.004	0.593
Education	−0.011	(−0.089, 0.066); 0.039	−0.033	0.001	0.771
Annual household income	0.042	(−0.020, 0.104); 0.031	0.157	0.025	0.184
Infant characteristics					
Gestational period	0.012	(−0.035, 0.059); 0.023	0.059	0.003	0.617
Birth weight	0.000	(−0.000, 0.000); 0.000	0.131	0.017	0.259
Age	−0.001	(−0.003, 0.000); 0.001	−0.260	0.068	0.022
Exclusive breastfeeding	0.013	(−0.201, 0.228); 0.107	0.014	0.000	0.901
Final multivariate model*				0.232	0.005
Maternal characteristics					
Age	0.000	(−0.014, 0.013); 0.007	−0.006		0.955
Current BMI	−0.017	(−0.043, 0.008); 0.013	−0.144		0.181
Use of DHA supplements	0.162	(0.035, 0.290); 0.064	0.277		0.013
Grilled fish intake	0.006	(0.000, 0.011); 0.003	0.234		0.036
Infant characteristics					
Birth weight	0.000	(−0.000, 0.000); 0.000	0.188		0.084
Age	−0.001	(−0.002, 0.000); 0.001	−0.196		0.086

^1^ *n* = 78. Linear model considering DHA in breast milk as the dependent variable and the factors listed as the independent variables. *Adjusted models were developed using DHA supplementation as a fixed effect.

## Discussion

This cross-sectional study was an early analysis of data from the Japanese Human Milk Study longitudinal birth cohort. The main strength of the study was to describe the effects of dietary patterns, including the use of DHA supplements, on milk FA compositions in the Japanese population. Analysis of the FA composition suggested a possible decrease in milk DHA concentration (median of 0.62% and mean of 0.65% for the entire population). This was hypothesized to reflect a decrease in seafood consumption among Japanese women (Table 1) in comparison with those in previous reports [which recorded mean milk DHA concentrations of 0.99% (6) and 1.09% (19)]. The milk DHA concentration closely matches the latest result from mature milk, in mothers of Japanese preterm infants born at <31 weeks of gestation [median of 0.63% (20)]. Although the milk sampling methods and study settings could influence such differences among studies, dietary patterns appeared to be the important factor to discuss regarding milk DHA concentrations. The maternal n–3 PUFA intake was in line with the latest DRIs in Japan (18); notably, 31% of the lactating women in this study routinely took dietary DHA supplements. To address the problem of FFQ-based estimation of DHA intake not including the use of DHA supplements, we investigated the effects of diet and use of supplements on milk FA composition in this study. We found no significant differences in the baseline characteristics of mothers or infants (Table 1) or in the macronutrient and energy content of breast milk according to DHA supplementation status (Table 2). Furthermore, there was no significant difference in maternal dietary intake according to DHA supplementation status (Table 1). However, the milk DHA concentration was higher in supplement users than in never-users (**Figure 1**); this may be attributed to the use of DHA supplements.

Consumption of fatty fish/seafood is another variable that potentially increases the milk DHA content. However, in our multivariate models, only consumption of grilled fish was positively associated with milk DHA concentrations after adjustment for use of DHA supplements. Although this finding was unexpected, it is consistent with the finding in a large-scale population-based study, that grilling is the most common way of cooking fish in Japan (21). Grilled fish may be the type most representative of the consumption of DHA-containing fish in younger Japanese populations. We estimated the DHA intake from the FFQ based on the seafood consumption in “the previous month,” whereas the milk DHA was determined by the “7-d” pooled milk. The difference in the mean duration of intake for the 2 variables might diminish the possible connection between the DHA intake and milk DHA. Because the BDHQ is an established method in Japanese populations to estimate the intake of foods and nutrients, we adopted the last month recall for the FFQ in this study. However, shorter estimates, such as 24-h recall, are possible options to clarify more direct relations between DHA intake and milk DHA concentration.

In contrast, DHA supplementation during lactation was likely to influence the milk DHA concentration in a frequency-dependent manner (Figure 1, Table 3). From the viewpoint of dosage, based on the linear regression of DHA + EPA supplementation ranging from 225 + 90 mg to 900 + 360 mg, a daily DHA + EPA intake of 1000 mg would be needed to reach a milk DHA + EPA concentration of 1% with no effect on the milk arachidonic acid content (22). Maternal dietary DHA and EPA content during the third trimester may affect the FA composition in mature breast milk (23), and the DHA intake in the third trimester may be insufficient for maintaining an adequate amount of DHA in maternal erythrocyte membranes (24). Furthermore, most of the maternal DHA supplements used by women in this study contained 350 mg DHA, and were made from fish oil (data not shown); this appeared to be in accordance with the products available on the Japanese market. Therefore, it is likely that the concentration of DHA in breast milk would be affected more by the frequency of DHA supplementation than by the dose, with no effect on the milk EPA or arachidonic acid content. Because DHA absorption is affected by its structural nature, phospholipid-bound DHA can be considered for further analyses.

We also found an association between milk DHA concentrations and infant age (Table 3). This finding is in contrast with that of a larger Asian cross-sectional study, which found no correlation between the concentrations of DHA in mature human breast milk and infant age (7), as well as the results in a Canadian cohort (5). However, another study reported an increase in the concentration of n–3 PUFAs in milk during the first 14 d of lactation, which was followed by a decrease in the milk DHA concentration between days 14 and 28 (25). On balance, these findings suggest that changes in the FA content of breast milk are neither consistent nor straightforward. The supplement used is more popular in pregnancy than in lactation, and DHA is not mentioned in the nutrients from supplements in North America (26). It is also possible that lactating mothers are more likely to take DHA supplements during the early stages of infancy.

In this study, total SFA concentrations were consistent, and total MUFA concentrations slightly varied by DHA supplementation status. The n–7 MUFA content was found to be higher in DHA supplement users (Table 2). To our knowledge, there have been no previous reports on the relation between milk n–7 MUFA concentrations and DHA supplementation in the literature. Given that oleic acid (18:1n–9) is one of the most abundant FAs found in the diet and is present in high amounts in olive oil and canola oil (27), it is unlikely that the use of DHA supplements, which are generally derived from fish oil, would be associated with MUFA concentrations in breast milk. Further investigations are required to identify factors associated with milk MUFA concentrations. Conversely, we found that the concentration of dihomo-γ-linolenic acid in milk was lower, with borderline significance, in supplement users than in never-users. Moreover, milk concentrations of α-linolenic acid and DHA were lower and higher in this study than those reported in a larger cohort, respectively (5). Genetic variants of FA desaturases (28) and FA elongases (29) might also affect the milk n–3 PUFA concentrations in lactating Japanese women. Increase in DHA intake does not correct low milk DHA in Taiwanese women carrying a single nucleotide polymorphism increasing genetic risk of FA desaturases (30). Information about gene variants will help further understandings of the relation between milk DHA and DHA intake as well as milk FA content.

A possible limitation for the FA analysis in this study is the type of internal standard used. We used tridecylic acid (13:0) and margaric acid (17:0) FAMEs as the internal standards, whereas pelargonic acid (9:0) is the most often used internal standard in combination with nonadecylic acid (19:0) for the analysis of milk FAs (31, 32). However, undecylic acid (11:0) FAME has also been used as an internal standard in a recent report (33), and the concentrations of major medium-chain FAs, such as caprylic acid (8:0), capric acid (10:0), and lauric acid (12:0), are comparable between our results and those in the literature (5, 6, 20, 33). Owing to their highly volatile nature, SCFAs like butyric acid (4:0) and caproic acid (6:0) are less contained than medium-chain FAs, and are sometimes influenced by other experimental conditions. Thus, more specific analytical techniques (GC-MS and NMR) are necessary to determine the content of SCFAs, as reported in the literature (34).

In conclusion, routine use of DHA supplements and adequate intake of dietary DHA from grilled fish have considerable potential for increasing the DHA concentration in breast milk in Japan. Our results suggested the milk DHA concentration in Japanese mothers reflected current seafood consumption and increasing adoption of DHA supplementation. However, further follow-up investigations and intervention studies are needed, particularly during lactation, to determine the implications of DHA supplementation for infant development.

## Supplementary Material

nzaa105_Supplemental_File

## Notes

Supported by Bean Stalk Snow Co., Ltd. and Megmilk Snow Brand Co., Ltd.

Author disclosures: HMU, SH, J-iY, YT, and TK are employees of Bean Stalk Snow Co., Ltd. YS, RW, HM, MS, and HK are employees of Megmilk Snow Brand Co., Ltd. These entities sponsored the study, participated in constructing the study protocol, and provided the infrastructure, materials, and human resources for the analysis of samples and data. Employees of these entities also participated in the interpretation of the results and in manuscript writing. Bean Stalk Snow manufactures and sells infant formula and dietary supplements for pregnant and lactating women in Japan. Megmilk Snow Brand manufactures and sells infant formula in Japan, Taiwan, Hong Kong, and Malaysia.

Supplemental Figure 1 is available from the “Supplementary data” link in the online posting of the article and from the same link in the online table of contents at https://academic.oup.com/cdn/.

Data described in the article, code book, and analytic code will be made available upon request.

HMU and SH contributed equally to this work.

Abbreviations used: BDHQ, Brief-type self-administered Diet History Questionnaire; DHQ, Diet History Questionnaire; FA, fatty acid.
