# Supplementary material for: Association of DHA Concentration in Human Breast Milk with Maternal Diet and Use of Supplements: A Cross-Sectional Analysis of Data from the Japanese Human Milk Study Cohort
Source: Curr Dev Nutr. 2020 Jun 15;4(7):nzaa105. doi: 10.1093/cdn/nzaa105 (PMC7343538; doi:10.1093/cdn/nzaa105)

Association of Docosahexaenoic Acid Concentration in Human Breast Milk with Maternal Diet and Use of Supplements: A Cross-Sectional Analysis of Data from the Japanese Human Milk Study Cohort, Hiroshi M. Ueno

Online Supplementary Material

Supplemental Figure 1 Participant disposition and flow through the study.

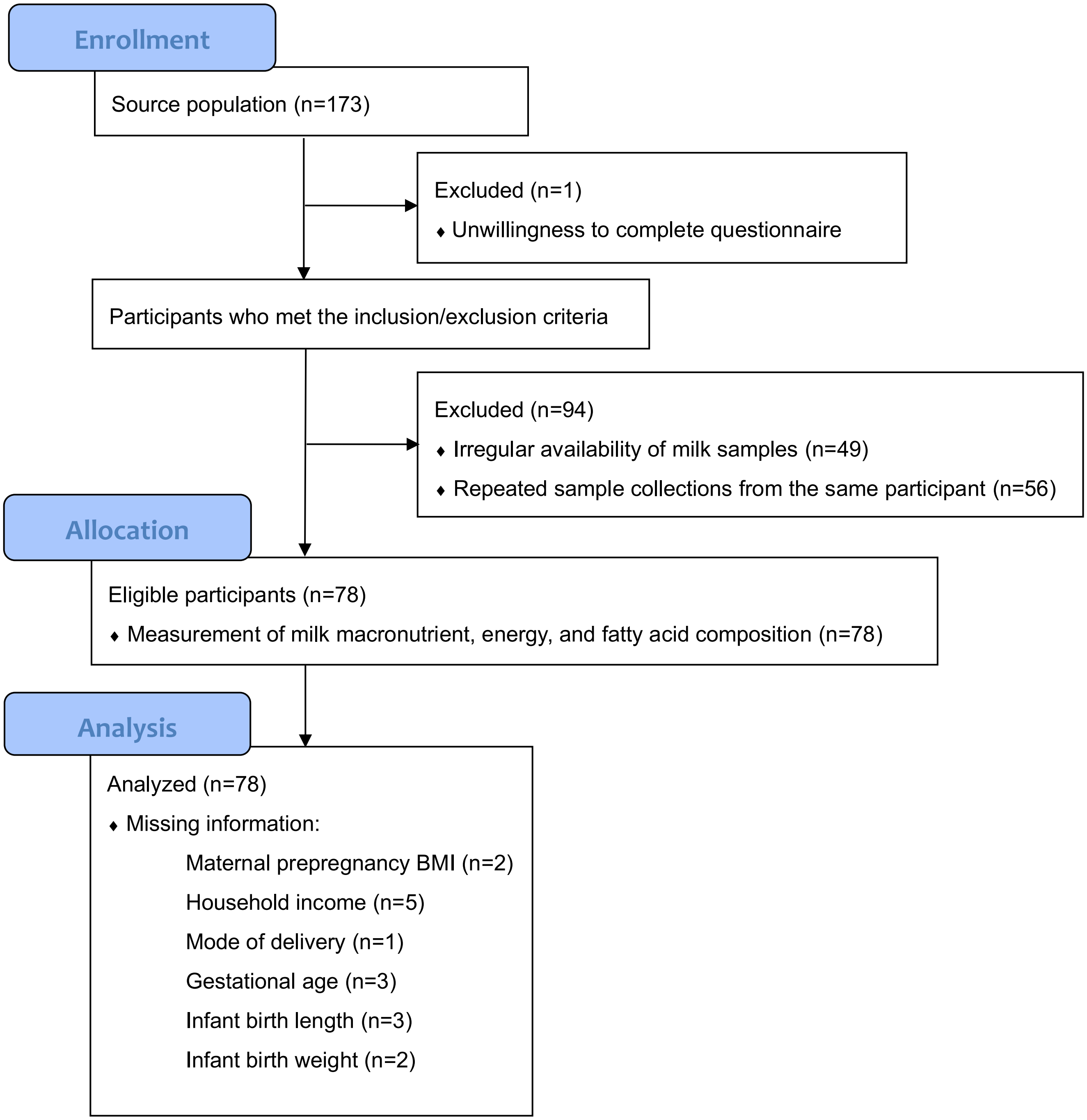

Supplement: nzaa105_Supplemental_File [file nzaa105_supplemental_file.pdf]
